# LncReg: a reference resource for lncRNA-associated regulatory networks

**DOI:** 10.1093/database/bav083

**Published:** 2015-09-10

**Authors:** Zhong Zhou, Yi Shen, Muhammad Riaz Khan, Ao Li

**Affiliations:** ^1^School of Information Science and Technology,; ^2^Centers for Biomedical Engineering and; ^3^School of Life Science, University of Science and Technology of China, 443 Huangshan Road, Hefei 230027, China

## Abstract

Long non-coding RNAs (lncRNAs) are critical in the regulation of various biological processes. In recent years, plethora of lncRNAs have been identified in mammalian genomes through different approaches, and the researchers are constantly reporting the regulatory roles of these lncRNAs, which leads to complexity of literature about particular lncRNAs. Therefore, for the convenience of the researchers, we collected regulatory relationships of the lncRNAs and built a database called ‘LncReg’. This database is developed by collecting 1081 validated lncRNA-associated regulatory entries, including 258 non-redundant lncRNAs and 571 non-redundant genes. With regulatory relationships information, LncReg can provide overall perspectives of regulatory networks of lncRNAs and comprehensive data for bioinformatics research, which is useful for understanding the functional roles of lncRNAs.

**Database URL**: http://bioinformatics.ustc.edu.cn/lncreg/

## Introduction

In the past few years, great progress has been made in the field of long non-coding RNAs (lncRNAs) (1, 2). Accumulated evidences show that lncRNAs play crucial roles in a wide variety of biological processes and in development and disease. Specifically, lncRNAs can affect apoptosis (3), cell differentiation (4, 5), autophagy (6), metabolism (7) and tumorigenesis (8). Many studies have demonstrated that lncRNAs can regulate gene expression through different mechanisms (9–12). For instance, *lincRNA-p21* (one of the highly studied lncRNAs) can regulate the expression of hundreds of genes at transcriptional level in *Mus musculus* by directly interacting with hnRNP-K (13). On the other hand, recent investigations discovered that lncRNAs can regulate mRNA translation through competing endogenous RNAs (ceRNA) activity (14–16), such as the relationship between *linc-MD1*, mRNA of *maml1* and *miR-133* (14). In addition, lncRNAs can influence protein stability at post-translational level. For example, hypoxia/HIF-1α induced *lincRNA-p21* can disrupt the interaction between HIF-1α and VHL in Hela cell, which results in HIF-1α accumulation (17). In general, lncRNAs can regulate protein functions or gene expression with different mechanisms: transcriptional regulation, post-transcriptional regulation, translational regulation and post-translational regulation.

Functional studies of lncRNAs are mainly based on high-throughput technologies and laboratory methods (18, 19). High-throughput technologies such as next-generation sequencing and microarray can provide plenty of functional information of lncRNAs. Laboratory methods such as quantitative Real-Time Polymerase Chain Reaction (qRT-PCR) and western blot are most widely used to validate the regulatory relationships and the functions of lncRNAs. However, when focusing on a specific regulatory relationship, data obtained from high-throughput technologies displays a high rate of false positives while data obtained from laboratory methods can provide precise mechanisms and accurate information. Therefore, data obtained from laboratory methods play a crucial role in understanding the functions of specific lncRNA.

Recent advances in technologies lead to a huge amount of information related to lncRNAs, which results in piling up of scattered literature. It’s not easy to obtain the scattered information from published literature. Therefore, along with the deep insight into the lncRNA community, many lncRNA-related databases have been built (20–26). For example, by collecting and analysing high-throughput ChIP-Seq data, Yang *et al*. (22) developed a database called ‘ChIPBase’ to decode transcriptional regulatory relationships of lncRNAs. Chen *et al*. (27) constructed LncRNADisease to store experimentally validated lncRNA-disease associations by manually reviewing the literature. These databases play important roles in the studies of lncRNAs.

Although attempts have been made to collect the scattered information about lncRNAs through the development of various databases, the comprehensive regulatory relationships between diverse lncRNAs and genes still remain ambiguous. To fill this gap, we developed LncReg database, which was short for ‘long non-coding RNAs regulatory database’, to collect lncRNA-related regulatory relationships. All entries in our database were manually assembled from published literature. The regulatory information about lncRNAs collected in this database has been validated by different experimental techniques such as qRT-PCR, western blot, ChIP, luciferase assay, etc*.* The users of this database will be able to gain regulatory information of a particular lncRNA, such as the targets of lncRNA, the key molecules involved in the regulation and the methods involved in the validation of lncRNA regulation. This will help researchers to understand the working mechanisms of lncRNAs and to design their experiments. With its user-friendly interface and valuable regulatory information about lncRNAs, LncReg will be a useful tool for biologists in the field of non-coding RNAs.

## Data sources and implementation

In order to cover regulatory relationships mediated by lncRNAs, abstracts of the articles related to lncRNAs were screened manually and then full text of selected articles was reviewed. Specifically, we used ‘long non-coding RNA OR lncRNA OR lincRNA’ as search parameters for PubMed (http://www.ncbi.nlm.nih.gov/pubmed/) on 20 December 2014 to obtain documents related with lncRNAs. The abstracts of our query were downloaded and manually screened to extract lncRNA-associated regulatory relationships. Next, full text of the selected articles was reviewed to capture the detailed regulatory information about lncRNAs. In order to provide comprehensive information, we integrated information about literature from other lncRNA databases, such as lncRNAdb (http://www.lncrnadb.org/) (28) and LncRNA2Target Database (http://www.lncrna2target.org/)(19).

The regulatory relationship entries in the database include lncRNA and gene information, regulatory aspects, species information, experimental evidences, detailed description of other parameters and reference information. Part of lncRNAs, which are deeply studied, also contain the information about key molecules involved in their regulation along with detailed mechanism. All the data in our database has been verified in published literature by laboratory methods, such as qRT-PCR and western blot.

The application architecture consists of a PHP presentation layer and MySQL persistent storage. Combined HTML/CSS and JavaScript enable an interface that is both easy to interpret and navigate. LncReg is supported by main standards-compliant web browsers such as Fire fox, Google Chrome, Internet Explorer and Safari.

## Content of the database

As shown in Table 1, 1081 lncRNA-associated regulatory relationship entries were culled manually from the literature recorded in PubMed. Two hundred fifty-nine articles were screened followed by full text review. There were 258 non-redundant lncRNAs and 571 non-redundant genes in LncReg. More detailed statistical information of LncReg and comparison with existing database is provided in Supplementary Table S1.

**Table 1.** Statistics information of LncReg database

Total entries	1081
Non-redundant lncRNA	258
Non-redundant gene	571
Upregulate	632
Downregulate	336
Active	27
Inactive	38
Transcriptional regulation	785
Post-transcriptional regulation	55
Translational regulation	23
Post-translational regulation	46

It is known that lncRNAs can influence genes expression by affecting the content or activity of gene productions. For example, *linc-MD1* can increase the protein levels of *maml1* and *mef2c* (14) and *H19* can increase the protein levels of *vimentin* (29). This regulatory relationship was recorded as upregulate in LncReg. Similarly, if the regulators inhibit the activity of the productions of target genes, such as the relationship between miRNAs and miRNA sponges (15, 30, 31), this regulatory relationship is recorded as inactive in LncReg. In the same way, two other types of regulatory relationships were labeled as downregulate and activate, respectively. Finally, we categorized the regulatory relationships into upregulate, downregulate, active and inactive accordingly. In LncReg, there are 659 positively regulated (632 upregulated and 27 active) and 374 negatively regulated (336 downregulated and 38 inactive) relationships. Furthermore, we developed four subsets based on regulatory mechanisms: transcriptional, post-transcriptional, translational and post-translational regulations. The lncRNA regulatory database LncReg contains 785 entries based on transcriptional, 55 on posttranscriptional, 23 on translational and 46 on posttranslational regulation.

The detailed information category of each entry has some preliminary tags. Each tag carries relevant information to the subsequent lncRNA. Regulators denote lncRNAs or genes which regulate other genes or lncRNAs and targets represent genes or lncRNAs regulated by relevant regulators. Most of the entries have detailed regulatory information including regulatory mechanism, validation methods and extended information. The validation methods consist of western blot, luciferase assay, qRT-PCR, RT-PCR, ELISA, immunofluorescence, RIP, ChIP, enzyme activity analysis, microarray and RNA-pull down, etc*.*

LncReg database has integrated other extended resources such as Gene Ontology information for further assistance of researchers. NCBI gene database can also be browsed directly from gene symbol. In addition, all the entries in the database can also be downloaded directly in Microsoft Excel format from the download tab. All the data on LncReg are freely available without any registration. The records of lncRNA-associated regulatory relationships are regularly updated by manually collecting from published literature. We will highly appreciate uploading of the results related to our database. The records submitted by individual user will be reviewed in time and entries will be added to the database after reviewing.

## Data querying and browsing

**Figure 1.**
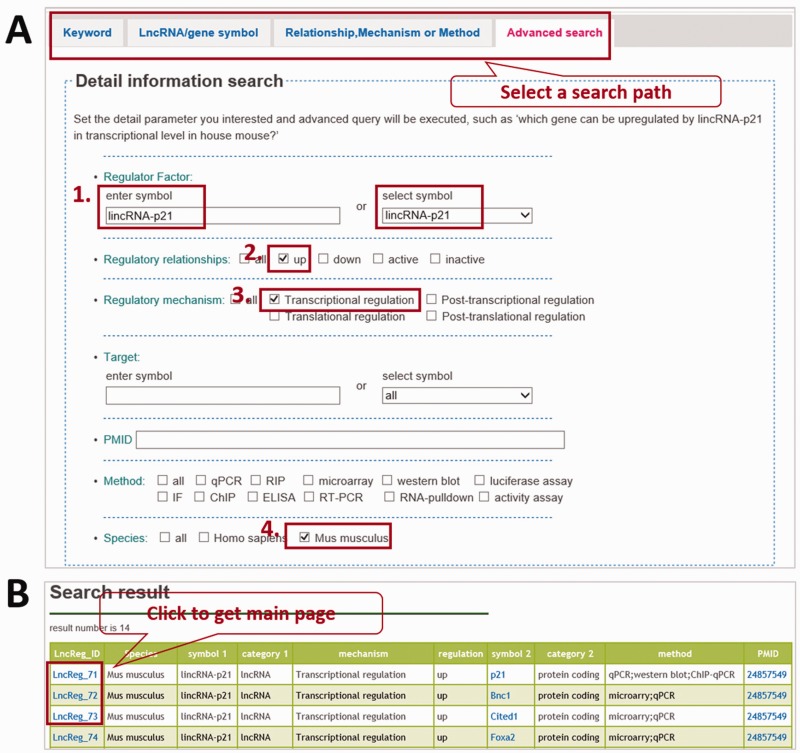
Querying LncReg database. (**A**) LncReg provides four searching tabs to users. To query the genes transcriptional upregulated by *lincRNA-p21* in house mouse, users can set parameters in four steps as shown in figure. (**B**) The display page of a representative query.

LncReg is a valuable tool for the researchers and has a user-friendly interface. There are two ways to explore the LncReg database: by searching a specific entry or by browsing. Four tabs are set to search a specific entry: search by keyword, search by lncRNA/gene symbol, search by relationship/mechanism/method and by advanced search. LncReg database will retrieve the data according to the query. Advanced search tab is a comprehensive tab to search particular entries based on multiple parameters. For example, users may be interested in genes upregulated by *lincRNA-p21* in house mouse. For this particular search, users can set ‘*lincRNA-p21*’ as regulator, ‘up’ as regulatory relationship, ‘transcriptional regulation’ as regulatory mechanism and ‘*Mus musculus’* as species (Figure 1A). LncReg database will show genes upregulated by *lincRNA-p21*.

Similarly, users can browse entries depending on classifications in ‘Browse’ page provided by LncReg. Furthermore, users can select a specific lncRNA in Browse page and a map/table that contains all the targets of this lncRNA and the regulation will be displayed in one go.

## Detailed data display

The display page of regulatory relationship entry for browsing or searching includes the symbols of lncRNA and gene, the categories of lncRNA and gene, species information, regulatory relationship, regulatory mechanism, validation method and PubMed Unique Identifier (PMID) of reference articles (Figure 1B). The NCBI gene information and PubMed literature can also be assessed through crosslink. The main page about a particular entry will be displayed by clicking the ‘LncReg_ID’ link.

**Figure 2.**
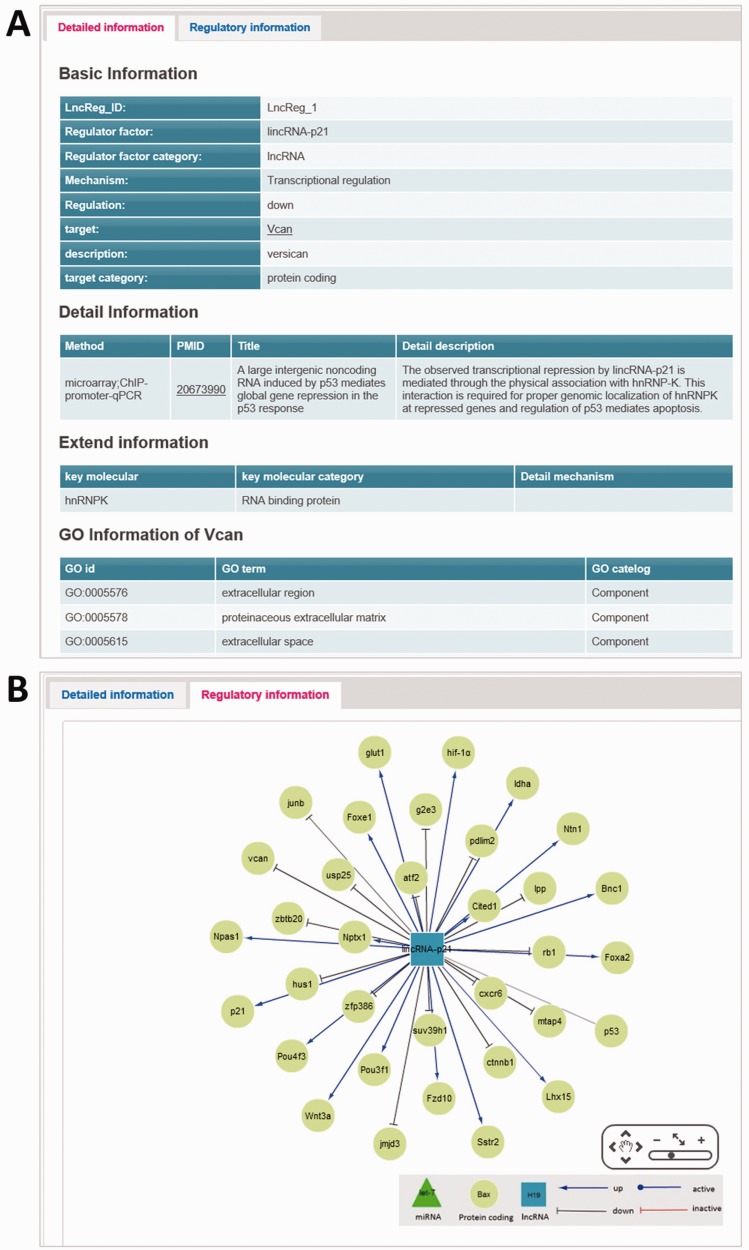
The main page of regulatory relationship entry. (**A**) The detailed information tab of main page. (**B**) The regulatory information tab of main page: representing the lncRNA-related regulatory network.

The main page of regulatory relationship entry has two tabs: detailed information tab and regulatory information tab. The first tab displays basic, detailed and extended information of a particular entry (Figure 2A). Moreover, this page also includes ‘GO information’ of the target gene. Basic information comprises of LncReg_ID, lncRNA and gene information, regulatory relationship and regulatory mechanism. The detailed information contains validation methods, PMIDs, titles of articles and detailed description. The ‘extended information’ incorporates the key molecules and detailed mechanism related to this lncRNA regulatory relationship if exists.

The second tab titled ‘regulatory information’ covers the regulatory network of regulator (Figure 2B). The purpose of this visualized network is to provide an overall perspective of the regulatory relationships of a specific regulator. Cytoscape Web (http://cytoscapeweb.cytoscape.org/), a powerful component that allows embed networks within HTML documents (32), was used to display the regulatory relationships. Regulatory relationships associated with a specific lncRNA can be displayed with different styles (for detail see the legend in website). Through the pan-and-zoom visualization architecture, users can also browse specific gene in the regulatory network.

## Discussion and future directions

The great achievements in prediction and research about lncRNAs (13, 33, 34) lead to the huge progress in the field of lncRNAs. With the intention to store lncRNA-associated information, many databases have already been developed (25, 26, 28) while regulatory relationships of lncRNAs are not highlighted. Here, through screening of published data manually, we collected validated lncRNAs associated regulatory relationships. These regulatory relationships were put together to build LncReg database. In the future, we expect to scan the literature automatically, a perfect way to maintain LncReg based on plenty of manually collected data and sophisticated text mining methods.

Accumulated evidences show lncRNAs can regulate gene expression through different mechanisms. For example, *lncRNA-p21* can regulate the transcription of plenty of genes involved in p53 pathway in *Mus musculus* (13) and *pint* has similar functions (35). Part of lncRNAs can regulate gene expression at post-transcriptional level, such as the regulatory relationship between *H19* and *let-7* (29, 36, 37). Meanwhile, other regulatory mechanisms, such as translational regulation of *lncRNA-p21* (38) and post-translational regulation between *lincRNA-p21* and HIF-1α(17), are also very important for the functions of lncRNAs. Accordingly, in order to provide the comprehensive information of regulations, we categorized the regulatory roles into transcriptional regulation, post-transcriptional regulation, translational regulation and post-translational regulation in LncReg.

Existing lncRNA-associated regulatory databases, such as LncRNA2Target, ChIPBase and starBase, provide a good deal of valuable resources of lncRNA (19, 21, 22). As shown in Table 2, those databases pay attention on different concerns of lncRNA-associated regulatory relationships. ChIPBase and starBase collect data obtained from high-throughput technologies and focus on transcriptional regulation of lncRNAs and RNA-associated interaction relationships respectively. LncRNA2Target is a useful database for differentially expressed genes after lncRNA knockdown or overexpression, which contains comprehensive data obtained from high-throughput technologies and laboratory methods. Compared with existing databases, LncReg is especially designed for lncRNA-associated regulatory relationships. Specifically, in order to collect regulatory relationships between lncRNAs and genes, the entries in LncReg are categorized into different types according to their regulatory relationships, i.e. upregulate, downregulate, active and inactive, respectively. Meanwhile, we categorize regulatory mechanisms into different types based on transcriptional, post-transcriptional, translational and post-translational regulations. Furthermore, LncRNA2Target collects information of genes regulated by lncRNAs while LncReg collects more comprehensive lncRNA-related regulatory information including not only genes regulated by lncRNAs but also lncRNAs regulated by genes. In addition, compared with LncRNA2Target, LncReg pays more attention on data obtained from laboratory methods that contain precise mechanisms and accurate information of regulatory relationships and contain more entries collected from laboratory methods (1081 vs. 396) that have been validated through various experimental methods, such as qRT-PCR, western blot, RNA-IP, ELISA, activity assay, etc. Finally, for further assistance of researchers, LncReg integrated functional information such as Gene Ontology information.

**Table 2.** Characteristics of LncReg and other lncRNA-associated regulatory databases

	ChIPBase	starBase	LncRNA2Target	LncReg
Role of lncRNA	Target	\	Regulator	Regulator/target
Relationship	\	\	\	Up/down/active/inactive
Mechanism	Transcription	Interaction	\	Transcription/post-transcription/translation/post-translation
Data type	High-throughput technologies	High-throughput technologies	High-throughput technologies and laboratory methods	Laboratory methods
Evidence	ChIP-Seq	CLIP-Seq	Knockdown/overexpression followed by qPCR/WB	qPCR, WB,RIP, IF, ELISA, activity assay *etc.*
Functional information	\	\	\	Gene ontology information

LncReg can provide regulatory information about lncRNAs, such as targets, regulatory mechanisms, experimental evidences for regulation and key molecules participating in regulation. Along with other functional databases of lncRNAs, LncReg will be a handy tool for biologists to retrieve lncRNA-associated regulatory relationships. At the same time, lncRNA-associated regulatory networks in our database provide a new overall perspective for functional studies of lncRNAs. Moreover, LncReg can provide high-quality comprehensive data to forecast innovative roles of lncRNAs for bioinformatics researchers (27, 39). This will help the users to explore the working mechanisms and functional roles of lncRNAs in detail and it will be helpful for the future studies of lncRNAs.

## Supplementary Data

Supplementary data are available at *Database* Online.

## Funding

This work was supported by grants from the National Natural Science Foundation of China (61471331 and 61571414). Funding for open access charge: Natural Science Foundation of China (61471331).

## Supplementary Material

Supplementary Data
