# Supplementary material for: LncReg: a reference resource for lncRNA-associated regulatory networks
Source: Database (Oxford). 2015 Sep 10;2015:bav083. doi: 10.1093/database/bav083 (PMC4565966; doi:10.1093/database/bav083)
Supplement: Supplementary Data [file supp_2015_bav083_index.html]

LncReg: a reference resource for lncRNA-associated regulatory networks — Supplementary Data 

# LncReg: a reference resource for lncRNA-associated regulatory networks

## Supplementary Data

files

- Supplementary Data - docx file
