# Supplementary material for: LncReg: a reference resource for lncRNA-associated regulatory networks
Source: Database (Oxford). 2015 Sep 10;2015:bav083. doi: 10.1093/database/bav083 (PMC4565966; doi:10.1093/database/bav083)
Supplement: Supplementary Data [file supp_bav083_additional_files.docx]

**Supplementary materials**

**Supplementary Table 1** Statistical information of data obtained from laboratory methods in LncReg and LncRNA2Target.

|  | LncReg | LncRNA2Target |
| --- | --- | --- |
| Total entries | 1,081 | 396 |
| Entries of genes regulated by lncRNAs | 1,017 | 396 |
| Entries of lncRNAs regulated by genes | 64 | \ |
| Nonredundant lncRNA | 258 | 94 |
| Nonredundant gene | 571 | 311 |
